# Identifying the Epitope Regions of Therapeutic Antibodies Based on Structure Descriptors

**DOI:** 10.3390/ijms18122457

**Published:** 2017-11-24

**Authors:** Jingxuan Qiu, Tianyi Qiu, Yin Huang, Zhiwei Cao

**Affiliations:** 1 School of Life Sciences and Technology, Tongji University, Shanghai 200092, China; 0107jxqiu@tongji.edu.cn (J.Q.); huangyinok@163.com (Y.H.); 2 School of Medical Instrument and Food Engineering, University of Shanghai for Science and Technology, Shanghai 200093, China; 3 The Institute of Biomedical Sciences, Fudan University, Shanghai 200433, China; 1132980@tongji.edu.cn

**Keywords:** immunogenic antibody, antibody humanization, structure descriptor, epitope prediction

## Abstract

Therapeutic antibodies are widely used for disease detection and specific treatments. However, as an exogenous protein, these antibodies can be detected by the human immune system and elicit a response that can lead to serious illnesses. Therapeutic antibodies can be engineered through antibody humanization, which aims to maintain the specificity and biological function of the original antibodies, and reduce immunogenicity. However, the antibody drug effect is synchronously reduced as more exogenous parts are replaced by human antibodies. Hence, a major challenge in this area is to precisely detect the epitope regions in immunogenic antibodies and guide point mutations of exogenous antibodies to balance both humanization level and drug effect. In this article, the latest dataset of immunoglobulin complexes was collected from protein data bank (PDB) to discover the spatial features of immunogenic antibody. Furthermore, a series of structure descriptors were generated to characterize and distinguish epitope residues from non-immunogenic regions. Finally, a computational model was established based on structure descriptors, and results indicated that this model has the potential to precisely predict the epitope regions of therapeutic antibodies. With rapid accumulation of immunoglobulin complexes, this methodology could be used to improve and guide future antibody humanization and potential clinical applications.

## 1. Introduction

Antibodies are a group of proteins that can specifically bind to different classes of target antigens through epitope regions [1]. The high specificity of this binding to special biomarkers or disease targets makes them ideal for disease diagnose and treatment [2]. For example, anti-procalcitonin antibody can be used to detect bacterial infection [3] and anti-PD1 antibody is currently used for cancer treatment [4]. Initially, therapeutic monoclonal antibodies (mAb) were produced by hybridoma cells which were generated by the fusion of spleen cells from mice immunized with an antigen of interest and myeloma cells [5]. Later, in clinical trials, researchers found that the human immune system would be alerted by those therapeutic mAb and produce anti-drug antibody (ADA) [6]. This meant that mAb were identified as exogenous antigens by the human immune system and neutralized by ADA. Thus, the therapeutic effect of those immunogenic antibodies (iAb) was limited. Moreover, if those ADAs can cross-react with human endogenous proteins, it may lead to serious autoimmune diseases [7]. In that case, reducing the immunogenicity of exogenous therapeutic antibodies is essential for maintaining the curative effect in antibody-dependent disease treatment. 

To achieve this goal, the most widely used method is called antibody humanization [8,9,10,11]. Previous studies have shown that the residue sequence of exogenous antibodies and human antibodies were quite different [9,10], and antibody humanization was aimed to reduce the immunogenicity by replacing sequences of exogenous antibodies with the corresponding part of a human antibody [8,11,12]. According to the structure of antibody, the variable region was the binding region for antigen–antibody interaction, and the consistent region, which was not involved in interaction, can be replaced by human antibodies (hAb). Through this process, the engineered antibodies can be defined as chimeric antibodies [13,14]. Later, the major binding region was further specified as the complementarity determining regions (CDRs) on antibody. In that case, to further reduce the immunogenicity of exogenous antibodies, only the CDRs were retained and the remaining framework regions (Fr) were replaced by human antibodies, resulting in humanized antibodies [15,16]. In 2005, Foote et al. compared the anti-drug resistance of several different antibodies, including murine antibodies, chimeric antibodies, and humanized antibodies [17]. In this research, different types of antibodies were injected into patients and results showed that marked ADA was detected in 9% of humanized antibodies, which was significantly less than those of murine antibodies (84%) and chimeric antibodies (40%). In 2008, Makabe et al. found that by grafting the CDR of murine antibody m528 to a humanized antibody scaffold, the antibody’s affinity for its target was reduced to 2.5% [18]. Results showed that the residue changes in Vernier zone can also affect the structure of CDR. This phenomenon was also confirmed by Foote and Winter, which indicated that the back mutation of Vernier zone can increase binding affinity [19]. Beside CDR graft, another strategy for antibody humanization is antibody resurfacing [20,21,22,23]. Through several residue mutations on antibody surfaces, resurfacing can maintain specificity and binding affinity [24]. The immunogenic residues for those resurfacing methods were detected in sequence level. With the development of biotechnology, humanized antibodies produced by TransChromo animals, which incorporate the entire human immunoglobulin loci, have been used in drug development [25,26]. Yet, even antibodies humanized in this way sometimes cause immunogenicity [27]. Thus, in addition to the humanization degree, the correlation between immunogenic antibody and ADA should also be considered for antibody engineering. In that case, methods which could consider those spatial features and precisely detect those residues with immunogenicity are highly desirable.

There are several methods available to detect the epitope region of antibodies [28,29]. For example, Li et al. develop a tool named OptMAVEn [28] including a human string content (HSC) score [30] to calculate the humanization level of every 9-mer-sequence derived from an antibody, based on the assumption that antibodies with more human sequence are expected to have lower immunogenicity for the human immune system [31,32]. Thus, antibodies containing more human sequences will have a reduced immunogenic potential. Notably, most of the spatial epitopes recognized by antibodies are comprised of several segments which are discontinuous in sequence but close in 3-D conformation [33,34], rather than simple linear sequences.

In this study, a dataset containing epitope regions of immunogenic antibodies was derived from iAb-ADA complexes. A series of analyses were then performed on those data to indicate the difference between antibody epitope regions and common antigen epitopes. Furthermore, the residual characteristics of epitope regions on immunogenic antibodies were detected. Following the above analysis, a computational model was established to predict epitope regions on antibodies. Results showed that the residual features derived from this article can be used to distinguish the epitope regions and non-immunogenic regions of antibodies. This study aimed to systematically detect the intrinsic features of conformational epitope regions in therapeutic antibodies and is expected to provide guidance for future antibody engineering.

## 2. Results

### 2.1. Difference Exists between Antibody Epitope Regions and Common Antigen Epitope Areas

Immunogenic antibodies and common antigens are both recognized and neutralized by the human immune system. However, as proteins with consistent structures and specific functions, antibodies contain epitope regions which might be different from common epitopes. In that case, the application of current established models for epitope prediction would be limited. To test the performance of spatial epitope prediction tools on immunogenic antibodies, SEPPA 2.0 [35] was selected for validation. In SEPPA 2.0 (spatial epitope prediction server for protein antigens version 2.0) , input antigens can be divided into nine categories according to its immune host and subcellular localization. In this study, as all ADAs were derived from humans, the immune host were selected as Homo sapiens and the subcellular localization was set as secreted or others. In that case, two different models were generated for prediction. Results showed that the best prediction ability of SEPPA 2.0 on those antibody epitopes can only reach a sensitivity of 0.514, which is extremely lower than its performance on common epitopes (0.797). Thus, the epitope features of immunogenic antibodies might be different from those of common antigens.

**Figure 1:**
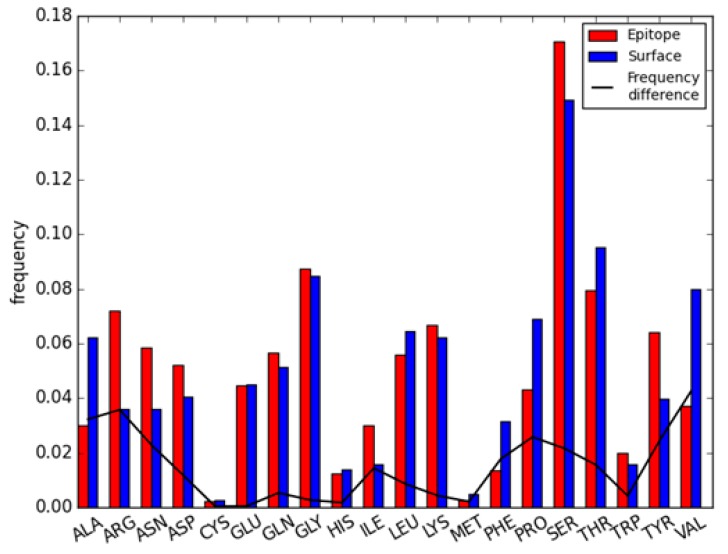
Amino acid distribution of both epitope region and surface non-immunogenic region on antibody.

In this article, 44 iAb-ADA complexes with 54 epitope regions (Table S1) were analyzed; all residues on antigen proteins can be divided into core residues, surface non-immunogenic regions, and epitope residues. The frequency of all 20 amino acids on those 54 epitope regions compared with corresponding non-immunogenic regions can be found in Figure 1. Red and blue bars represent residue frequency on epitope regions and surface non-immunogenic regions respectively; while the black line is the frequency difference between two regions. The occurrence of amino acids on different regions were as follows: ARG, ASN, SER, and TYR were preferred in epitope regions while ALA, PRO, and VAL were preferred in surface non-immunogenic regions. Among those, VAL and ALA are hydrophobic residues, ARG is a basic residue and TYR is an aromatic residue. The amino acid preferences are quite similar to Sun’s study on common antigen epitopes [36], which indicate TRP, TYR, ARG, and HIS were more preferred in epitope regions, while CYS, ALA, and VAL had lower frequencies. However, the amino acid frequencies in the epitope regions of immunogenic antibodies were different in comparison to epitopes from conventional protein antigens. For example, SER was the most enriched amino acid with a frequency over 14% in both epitope and non-immunogenic surfaces of antibodies, far above the random frequency of 20 amino acids (~5%), but the enrichment of SER is below average in common protein antigens [36]. Conversely, MET and TRP were enriched in the epitope regions of common antigens, but not in the epitope regions of antibodies. The above results indicate that even though residue preferences are similar between the epitope regions of common antigen and those of antibodies, the enrichment patterns are quite different. For example, hydrophilic amino acids (such as SER) are enriched in antibodies, and hydrophobic amino acid (such as TRP) are enriched in common antigens. This will lead to different features of both structure and physio-chemical properties. In that case, deriving features to characterize antibody epitopes and establishing a computational model for antibody epitope prediction is desirable.

### 2.2. Residue Character of Epitope Region on Immunogenic Antibodies

**Figure 2:**
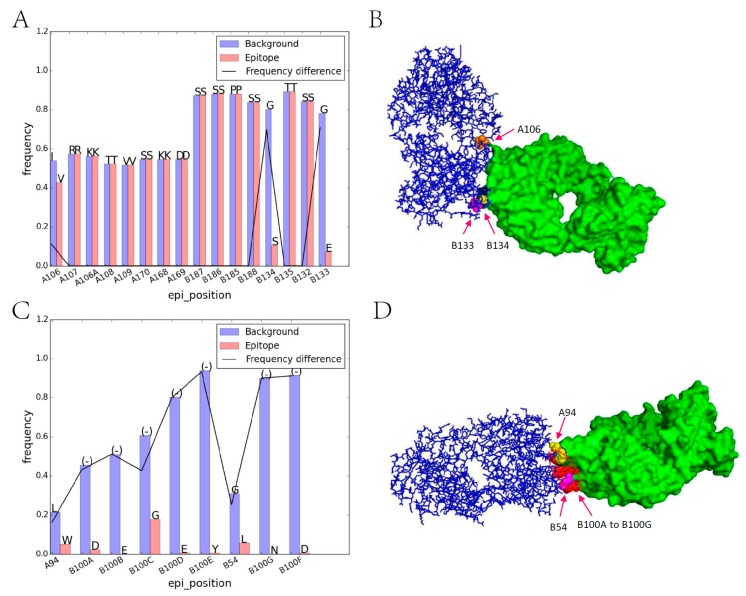
Illustration of amino acid substitution and insertion on iAb epitope regions. (**A**) Residue frequency comparison between iAb epitope (PDB id: 1AD9) and background, letters above each bar represents the abbreviation of different amino acids; (**B**) Corresponding crystal structures of iAb-ADA interactions. iAb is marked in blue while ADA is marked in green. The substitutions in subgraph **A** are labeled as orange (A106), pink (B133), and yellow (B134) in subgraph **B**, respectively; (**C**) Residue frequency comparison for 1RZ8; (**D**) Corresponding crystal structures. The residue insertion in subgraph C are marked in red (B100A to B100G) and the substitutions are marked in yellow (A94) and pink (B54), respectively. PDB: protein data bank; iAb: immunogenic antibodies; ADA: anti-drug antibodies.

In order to discover the residue character of epitope regions on immunogenic antibodies, the amino acid composition on corresponding sites were firstly analyzed. Among all 44 iAb-ADA complexes, the light chain and heavy chain of 42 complexes were clearly defined and can be compared with those 178 nonbinding human antibodies. After sequence alignment, the corresponding positions of 220 light chains and 220 heavy chains were aligned, and the amino acid distribution on each position can be compared between those iAbs and background antibodies. In this case, the most frequently occurring residue in the background antibody was selected to compare with the residue on the corresponding position of iAb. Of the 803 epitope positions, 445 (55.42%) positions contained the same amino acid and 358 positions differed. That means that more than half of the epitope positions were without residue variation. Further analysis shows that different situations occurred in different epitopes. In our dataset, most of the epitopes (91.8%) involve residue substitution with big physio-chemical environmental variations. For example, in Figure 2A, the epitope region contains three substitutions, both B133 and B134 substituted from the most frequent GLY to SER and GLU, respectively. These results indicated that the micro-environment variation may alert the immune system and for recognition by ADA. Another notable characteristic is residue insertion; more than 48.9% of the epitopes involve residue insertion (Figure 2B), and several epitopes contain both mutations and insertion. Also, a few epitopes (4.1%) contain no mutations and residue insertions, which means epitope regions may also have been affected by neighboring environments around epitope regions.

As the functional domain, complementarity-determining regions (CDR) of protein antibodies are essential for antigen recognition and binding. Hence, if the CDRs were recognized as an epitope region by the immune system, the therapeutic antibody will lose efficacy. According to Kabat’s numbering scheme, CDRs were defined as heavy chain numbers 31–35b, 50–65, 95–102, and light chains 24–34, 50–56, 89–97 [37]. After multiple sequence alignment (MSA), all antibodies were aligned. If one epitope region contains over 50% of the CDR residues, this epitope regions will be defined as a CDR-based epitope. In our dataset, over 46.9% of the epitope regions can be defined as CDR-based epitopes, among them, over 86.9% of the epitope regions contain residue insertions. The analyses described above, suggest that the antigenicity variation in the antibody surface results from amino acid substitution and insertion.

To further demonstrate the relationship between the properties of amino acids and the character of epitope regions, the physiochemical indices derived from AAindex [38] were used for quantitative characterization between epitope regions and non-immunogenic surfaces. Six physiochemical properties which were essential for antigen–antibody interaction were tested, including hydrophobic index, localized electrical effect, number of hydrogen bond donors, normalized van der Waals volume, hydrophobic parameter, and isoelectric point. For each spatial epitope, the average AAindex scores for one physiochemical property were calculated on background antibodies and compared with the same regions in immunogenic antibodies. Using a two-tailed *t*-test, three indices with significant difference (*p* value < 0.05) were selected (Table 1). It was determined that the amino acids with contrasting properties (such as van der Waals volume, H-bond, hydrophobicity, and isoelectric) will alter the microenvironment of the surface of the antibody.

### 2.3. Model Construction and Performance

Through the analysis described previously, it was determined that the antigenicity variation in antibody surfaces was mostly caused by residue substitutions and insertions. Furthermore, the protein shape, physiochemical properties of the micro-environment, and accessibility of surface areas of the antibody would be changed. In that case, those three features will be used to generate our residue descriptors. Firstly, for each residue, its Euclidean distances from the center of two disulfide bonds were calculated and generated as two-dimensional descriptors. Secondly, the physiochemical micro-environment change of each residue can be generated through a shell structure model. Thirdly, the accessible surface areas of each residue can be calculated by Naccess (Version 2.11). Finally, all of the features described above were integrated into a 151-bits descriptor. After descriptor generation of each surface residue, a logistic regression was used to generate a classification model.

**Figure 3:**
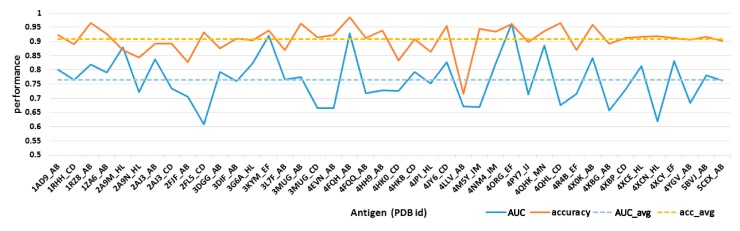
Model evaluation through ten-fold cross-validation. Solid lines refer to the area under roc curve (AUC) value and prediction accuracy for each immunogenic antibody while dashed lines refer to the averaged AUC and accuracy (acc).

For each antibody in our dataset, every surface residue can be transferred into a 151-bits descriptor and will be predicted as epitope residues or non-immunogenic surface through logistic regressions. Then, ten-fold cross-validation was used for model evaluation. The classification accuracy and area under roc curve (AUC) value can be found in Figure 3. It can be seen that the accuracy for most structures ranged from 0.72 to 0.97, with an average accuracy of 0.9. The AUC value ranged from 0.6 to 0.95, with an average AUC value over 0.765. Furthermore, all epitope residues and non-immunogenic surface residues from 42 structures were integrated to establish a classification model. Results showed that our model can achieve a good performance with AUC value over 0.731 and accuracy over 0.949.

## 3. Discussion

The humanization of therapeutic antibodies has long attracted the interests of researchers due to their potential application in treating important diseases. Since the drug efficacy of those antibodies synchronously decreases as more exogenous sequences are replaced by human antibodies. Hence, the prediction of epitope regions on antibodies will guide residue mutations and minimize antibody modification. However, antibody-antibody interaction is a complex process which is different from protein-protein interactions and even the common antigen-antibody interaction. That means epitope prediction of common antigens is not applicable to antibodies, which blocks further progress of antibody humanization. In this study, a representative dataset has been collected to extract differences in epitope regions and non-immunogenic surface on exogenous antibodies.

Antibodies are protein macromolecules. When antibodies are detected as protein antigens by the human immune system, the epitope regions should share features with common antigens. For example, the amino acid preference between epitope regions and non-immunogenic surfaces for immunogenic antibodies are quite similar as those of common antigens. However, as a soluble secretory protein with special functions, the amino acid preference between immunogenic antibodies and common antigens are not the same. Also, the surface form of immunogenic antibodies might be different from those of common antigens, since the epitope regions of common antigens showed different shapes for antibody binding, but antibodies all maintain “Y” shaped structures for specific functions. That might explain why methods designed to predict epitope regions on common antigens are not applicable for immunogenic antibodies.

Furthermore, immunogenic antibodies are aligned with background antibodies to demonstrate the features of epitope regions on antibody surfaces. Results showed that two factors may change the antigenicity of the antibody surface and make it an epitope area recognized by the immune system. One is amino acid substitutions with significantly physiochemical property changes, such as substitution of alkaline and hydrophobic residues with hydrophilic and sulphur-containing residues or others. Another issue is amino acid insertions; such insertions happened in CDR. Normally, insertions containing residues with essential physiochemical properties such as hydrophobicity, hydrogen bonds and van der Waals forces will promote the interaction between antibodies. It can be noticed that in our dataset, no deletion was discovered for those immunogenic antibodies in CDR.

After comprehensive comparison of epitope and non-immunogenic surface, and epitope and corresponding background area, the distinguishable features of epitope regions on antibodies were summarized to establish a computational model. Because of the limitation of data accumulation, this model was only evaluated through internal validation. However, the performance of this model illustrated the reliability of those features, and indicated the potential of this model for future applications. Apart from B cell prediction, T cell epitope prediction was also conducted as the initial step for de-immunization of therapeutic antibodies [39]. According to the above results, potential immunogenic amino acids could be substituted to reduce ADA, while the stable structure of the antibody is maintained. Combined with different humanization strategies, which allow an introduction of a high proportion of human antibody sequence, elimination of potential immunogenic epitope residues can be used to optimize an antibody for therapeutic applications.

## 4. Materials and Methods

### 4.1. Data Source

All protein structure data used in this article were derived from Protein Data Bank (PDB) [40]. With keywords of “antibody”, 2434 PDB ids were initially collected. Among those, 545 PDB ids containing interaction between human antibody and other antibodies remained. From them, 44 iAb-ADA complexes (Table S1) and 197 human antibody monomers (Table S2) were manually screened as our dataset. For each PDB complex, the epitope regions were determined as those residues with the nearest atom distance to the other side (less than 5 Å). In our dataset, three types of epitope regions were discovered (Table S3): (1) located in constant region (Fc), (2) located in the Fab region (non-CDR), and (3) located in variable region (Fv).

### 4.2. Disulfide Bond-Based Spatial Location Descriptor

**Figure 4:**
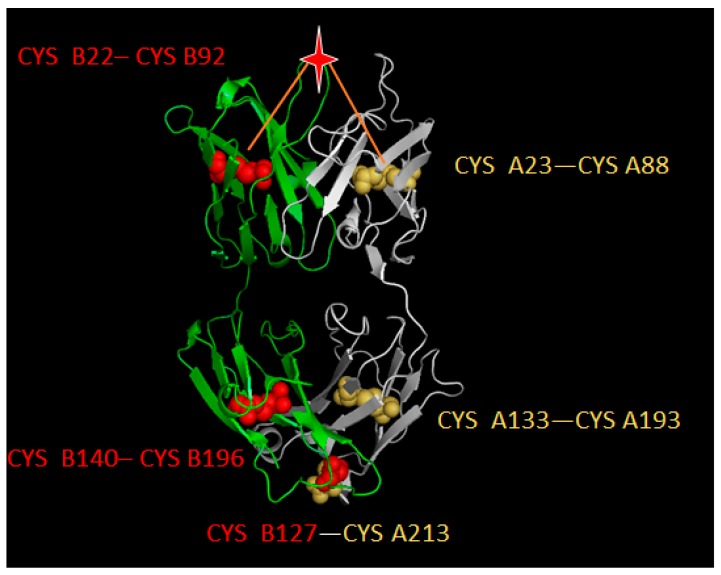
Disulfide bond illustration on the three-dimensional structure of an antibody (PDB id: 1AD9). The light chain (chain A) was marked in white while the heavy chain (chain B) was marked in green. Balls show the location of CYS and the star refers to the target residue to be described. Red balls represent CYS on heavy chain while yellow balls represent CYS on light chain. Red lines illustrate the Euclidean distance from benchmark points.

Unlike common protein antigens, antibodies have relatively consistent structures with two heavy chains and two light chains to form a “Y” shape structure. The crystalized structure of antibody binding fragment (Fab) is showed in Figure 4. It can be seen that antibodies contain disulfide bonds (CYS–CYS) in order to make the structure stable. In the Fab part, both light and heavy chains contain two disulfide bonds (light chain: A23–A88, A133–A193; heavy chain: B22–B92, B140–B196 respectively). Furthermore, another disulfide bond (B127–A213) was formed to link two chains. Here, two geometric centers of each disulfide bond in the variable region were selected as benchmark points and the Euclidean distance between target residues and two benchmark points were calculated to generate a two-dimensional descriptor.

### 4.3. Spatial Environment Descriptor through Neighboring Layers

For each target residue R, all its neighboring residues were clustered into different layers according to the Euclidean distance from R. In this study, all residues within the neighborhood (<20 Å) of R were derived and clustered into ten layers (2 Å for each layer). Indices including hydrophobicity index (ARGP820101) [41], number of hydrogen bond donors (FAUJ880109) [42], and normalized van der Waals volume (FAUJ880103) [42] of neighbouring residues were summarized in different layers as spatial environment descriptors for physiochemical description.

### 4.4. Surface Exposure Descriptor

Previous studies indicated that residues located in the surface with relatively prominence provide essential affections for protein-protein interactions. Here, the accessible surface area (ASA) value was used to illustrate the prominence of each residues and been calculated by Naccess (Version 2.11) [43].

### 4.5. Integrated Descriptors for Structure Information and Physio-Chemical Properties

**Figure 5:**
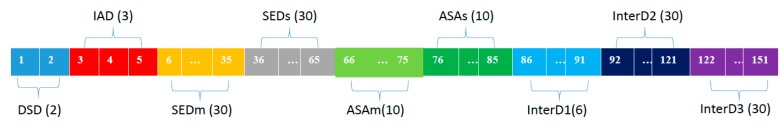
Illustration of structure descriptors. For each residue, a 151-bits structure descriptors were generated to characterize the structural features of target residues and their neighboring environment. DSD represents disulfide bond-based spatial location descriptors; IAD represents individual AAindex descriptors; SEDm represents spatial environment descriptor-max; SEDs represents spatial environment descriptor-sum; ASAm represents ASA value-max; ASAs represents ASA value-sum; InterD1 represents integration of disulfide bond-based spatial location descriptors and individual AAindex descriptors; InterD2 represents integration of ASA value and AAindex-max; InterD3 represents integration of ASA value and AAindex-sum.

For each residue on the antibody surface, an integrated descriptor was generated to characterize the structure display and physiochemical properties (Figure 5). Including six simple descriptors and three cross-term descriptors. Simple descriptors contain the following aspects: (1) Disulfide bond-based spatial location descriptors (DSD): distance of each residues to fixed point; (2) Individual AAindex descriptors (IAD): physio-chemical properties for individual residue, involving hydrophobic, H-bond and van der Waals volume; (3) Spatial environment descriptor-max (SEDm): recording the maximum AAindex value in different layers. Here, three types of index and 10 layers make it 30-bits descriptors; (4) Spatial environment descriptor-sum (SEDs): recording the summarized AAindex value in different layers (10-bits); (5) ASA value-max (ASAm): recording the maximum ASA value in different layers (10-bits); (6) ASA value-sum (ASAs): recording the summarized ASA value in different layers. Cross-term descriptors contain 3 types: (1) integration of disulfide bond-based spatial location descriptors and individual AAindex descriptors (InterD1); (2) integration of ASA value and AAindex-max (InterD2): for each layer, multiple the maximum ASA value and AAindex value; (3) Integration of ASA value and AAindex-sum (InterD3) (for each layer, multiply the summarized ASA value and AAindex value).

## 5. Conclusions

In summary, a large-scale analysis has been done to discover the characters of conformational epitopes on immunogenic antibodies. The results of this paper showed that epitope regions showed differences between immunogenic antibodies and common antigens, and extracted essential features to distinguish epitope regions and non-immunogenic surfaces of therapeutic antibodies. Finally, a computational model was established based on those distinguishable features to predict the epitope regions on antibodies. The outputs of this study provide the basis for improving the future design of therapeutic antibodies with enhanced humanization.

## Figures and Tables

**Table 1:** Physio-chemical indices between epitope region and the corresponding background region.

AAindex	Property Description	*t*-Test *p*-Value
ARGP820101	Hydrophobicity index	0.018
FAUJ880108	Localized electrical effect	0.421
FAUJ880109	Number of hydrogen bond donors	0.002
FAUJ880103	Normalized van der Waals volume	2.0 × 10^−4^
LEVM760101	Hydrophobic parameter	0.404
ZIMJ680104	Isoelectric point	0.055

## Supplementary Materials

Supplementary materials can be found at www.mdpi.com/1422-0067/18/12/2457/s1.
