# Supplementary material for: Identifying the Epitope Regions of Therapeutic Antibodies Based on Structure Descriptors
Source: Int J Mol Sci. 2017 Nov 24;18(12):2457. doi: 10.3390/ijms18122457 (PMC5751102; doi:10.3390/ijms18122457)
Supplement: Supplementary file 1 [file ijms-18-02457-s001.pdf]

Supplementary Table 1. Dataset of 44 aAb–ADA Complexes with 54 Epitopes

PDB ID	aAb chains	ADA chains	Epitope
**1AD9**	A,B	H,L	A170,A168,A169,A109,A106,A107,A108,A106A,B187,B186,B185,B188,B134,B135,B132,B133
**1ADQ**	A	H,L	A436,A437,A434,A435,A433,A438,A422,A424,A428,A440,A384,A385,A386,A254,A255,A252,A253,A251,A311
**1RHH**	C,D	A,B	D131,D133,D134,C151,C205,D129,D128,C207,C206,C204,C203,C202,C189,C212,C213,C196,C194,C201,C209
**1RZ8**	A,B	C,D	A94,B54,B100A,B100B,B100C,B100D,B100E,B100F,B100G
**1ZA6**	A,B	G,H	A56,A98,B70,B71,A33,A32,A31,A36,A34,A38,A100,B100,B51,B58,B59,B50,B56,B57,B55,B60,B69
**2A9M**	H,L	I,M	H148,H141,H143,H142,H145,H144,H146,H10,H11,H12,H14,H9,H6,H7,H101,H103,H102,L49,L48,H47,H46,H48
**2A9N**	H,L	I,M	H141,H142,H145,H144,H146,H10,H11,H12,H6,H7,H26,H103,H3,L49,H5,H48
**2AJ3**	C,D	E,F	D131,D132,D133,D136,D189,D187,D186,C205,D129,D128,D127,D126,C207,C206,C210,C213,D190,C209,C208,D213
**2AJ3**	A,B	C,D	B127,B126,B129,B128,B190,A213,A210,A209,A208,A115,B187,B186,B189,B213,B217,B215,B214,A205,A207,A206,B136,B134,B132,B133,B131
**2FJF**	A,B	E,F	A94,A92,B31,B33,A32,A31,A30,B103,B102,B101,B100,B105,A28,A29,B59,B52,B53,A68,A67
**2FJF**	A,B	M,N	B29,B70,B71,B72,B73,B74,B30,B51,B58,B52,B56,B57,B54,B55,B80,B82,B85,B84,B19,B60,B67,B66,B65,B64,B69,B68
**2FL5**	C,D	H,L	D85,D83,D82B,C153,C157,C154,C185,C188,D66
**2J6E**	A,B	H,L	B443,A437,A438,A439,B418,B415,A350,B414,B361,B360,A420,A422,A424,B358,B359,B356,B354,B355,A444,A443,A442,A441,A440
**2J6E**	A,B	I,M	B442,B443,B440,B441,B444,B445,A356,A355,A359,A358,B422,B424,A354,A414,B350,B420,A443,A360,B439,B438,B437,A418
**3D69**	A,B	H,L	A50,A53,B97,A93,A49,B52A,A32,A31,A30,B58,B98,B52,B53,B56,B54,B55,B100A
**3DGG**	A,B	C,D	A54,A57,A56,A99,A98,A95,A94,A97,A96,B35,B31,B32,B33,A33,A32,A31,A30,A36,A34,B99,B47,A71,B103,B102,B101,B100,B104,B59,B52,B50,B57,B54,B55

**3DIF**	A,B	C,D	A95,A94,A93,A26,A27,B58,B59,B85,B63,B62,B61,B60,B66,B65,A1,A3,A2
**3G6A**	H,L	A,B	H18,H19,H10,H11,H12,H17,H156,H211,H210,H213,H212,H215,H214,H21,H20,H209,H80,H125,H76,H208,H8,H9,H7,H5
**3KYM**	E,F	A,B	F104,F103,F102,F101,F100,E91,E93,E92,E94,E50,F56,F57,F54,F53,F59,F99,F30,F31,F32,F33,E32,F74
**3L7F**	A,B	D,E	A96,A91,A93,A92,B105,A32,A31,A30,B104,A28,A68,A67,B60
**3L7F**	A,B	H,L	A78,A77,A76,A74,A72,A70,A20,A22,A64,A65,A66,A67,A63,A17,A16,A18
**3MUG**	A,B	E,F	A188,A208,A151,A150,A153,A152,A191,A190,A189
**3MUG**	C,D	E,F	C153,C152,C156,C154,C9,C8,C7,C6,C5,C4,C69,C197,C145
**4EVN**	A,B	I,J	B27,A53,A52,A54,A56,A58,B96,B94,B95,A99,A98,B31,B33,A31,A101,A52A,B67
**4FQH**	A,B	H,L	B52,B53,B50,B54,B60
**4FQQ**	A,B	H,L	A50,A53,A52,A54,A56,B99,B32,A49,A77,B100,A64,A60,A62,A63,B100M,B100A,B100B,B100C,B100D,B100E
**4HH9**	A,B	C,D	A50,A53,A52,A55,A54,A56,A59,A58,A49,A60,B1
**4HK0**	C,D	A,B	C44,C40,C41,C42,C43,D167,D166,D165,D162,D168,C91,C90,D8,D9,D6,D7,D5,C184,C185,C186,C183,D11,C88,C89,D145,D144,D143,D142,D101,D104,D106,D108,D174,D21,D23,D22
**4HKB**	C,D	A,B	C78,C73,C76,C21,C214,C217,C219,C9,C8,C7,C19,C12,C11,C10,C80
**4JPI**	H,L	A,B	L6,L5,L165,L164,L163,L161,L9,L158,L159,L157,L10,L145,L144,L147,L146,L149,L148,L27,L101,L100,L103,L102,H168,H43,H42,H41
**4JY6**	C,D	A,B	D153,D151,D156,D154,D155,D158,D199
**4LLV**	A,B	C,D	A100D,A100E,A100,B30,B32,A100C,A100A,A99,A98
**4LLV**	A,B	E,F	A73,A53,A54,A100C,A100B,A100A,A29
**4LLV**	A,B	H,L	A100D,A100E,B27A,B29,B27,A64,B1,B2,A65,A61,A51,A50,A52,A54,A57,A56,A59,A58,A47,A62,A100C,A100F,B94,B95,B92,B93,A33,A31,A35,A95
**4M5Y**	I,M	H,L	I52,I32,I100A,I30,I28,I27,I97,I99,I98,I100,I31
**4NM4**	I,M	H,L	I1,M55,M54,M57,M56,M50,M53,M59,M58,I100C,M49,M60
**4ORG**	E,F	H,L	E100,E24,E25,E26,E27,E28,E99,E98,E100F,E100E,E100D,E100A,E73,E76,E75,E74,E52A,E32,E31,E30
**4PY7**	I,J	A,B	J24,J20,J197,J8,J9,J5,J6,J7,J3,J145,J144,J143,J142,J141,J101,J100,J107,J12,J10,J11

**4QHK**	M,N	I,J	N49,M100,N56,N54,N52,N53,N50,N51,N29,N66,M99,M100B,M100A,N30,N31,N32
**4QHL**	C,D	A,B	D49,D52,D53,D54,D56
**4R4B**	E,F	A,B	F206,F207,F208,F1,F3,F4,F5,F7,F23,F27,F26,F25,F24,F28,F197,F198,F199,F75,F76,F77,F153,F155,F156,F157,F158
**4X0K**	A,B	H,L	B29,B28,B27,B27E,B27D,B27A,B27C,B69
**4XBG**	A,B	E,F	B77,B78,B79,B45,B59,B81,B80,B16,B15,B61,A1,B168
**4XBG**	A,B	J,K	B76,B77,B59,B54,B18,B61,B60
**4XBP**	C,D	H,L	D30,C31,D94,D92,D93,D1,C64,C61,C56,C54,C53,C52,C59,C58,D29,D27
**4XCE**	H,L	A,B	H100F,H100,H100D,H100E,L52,L54,L18,L65,L64,L66,L60,H98,H99,H100C
**4XCE**	H,L	C,D	H54,H55,H56,H57,H52,H58,H64
**4XCN**	H,L	C,D	L79,L45,H27,H26,H100,L57,L58,L81,L80,L61,H3,H1
**4XCY**	E,F	A,B	E95,E54,E56,E52,E100G,E100F,E100E,F94,F30,F32,E31
**4XCY**	E,F	J,K	E68,E65,E59,E55,E54,E57,E56,E53,E19,E17,E81,E79,E72,E70,E74,E82A,E82B
**4YGV**	A,B	F,L	A50,A52,A55,A54,A56,A59,A104,B31,B32,B33,A33,B99,A72,A102,A103,B101
**5ANM**	E,F	C,D	F341,F340,F431,F430,F433,E393,E392,E391,E390,E394,F419,F427,F428,F429,F342,F503,F437,F436,F435,F434,F432,F472
**5BVJ**	A,B	C,D	A53,A52,A54,A59,A58,A77,A76,A64,A65,A60,A61,A62,A63,A17,A16,A18
**5CEX**	A,B	C,D	B28,B27,B26,A50,A53,A52,A55,A54,A57,A56,A59,A58,B94,B31,B32,A49,A79,A77,B100,A60,A61,B1,B2,B100A,B100B,B100C

Supplementary Table 2. Dataset of 197 Human Antibody Monomers

PDB ID	PDB ID	PDB ID	PDB ID	PDB ID	PDB ID	PDB ID
1AD0	2F5A	3KYK	4F58	4LRI	4R7N	5CGY
1AXS	2FB4	3LRS	4FNL	4LRN	4RIR	5CHN
1B4J	2FGW	3MME	4FQ1	4M6M	4RNR	5CIP
1BEY	2G75	3N9G	4FQ2	4M6N	4TOY	5D6C
1CLY	2IG2	3NA9	4FQC	4M6O	4UOK	5D7S
1DFB	2JB5	3NAA	4FQL	4NPY	4UOM	5DK3
1DN0	2JB6	3NAB	4FZE	4NRY	4UT7	5DT1
1DQL	2PR4	3NAC	4G5Z	4NRZ	4UV4	5F6H
1FGV	2VXV	3NCJ	4GSD	4NUG	4X4X	5F6I
1FH5	2XZA	3NFS	4GXV	4NUJ	4X4Y	5F7E
1FVC	2XZC	3OAU	4HFW	4NYL	4X7T	5FHA

1FVD	2YK1	3P0V	4HIE	4NZU	4XCC	5FHB
1FVE	2YKL	3PIQ	4HIH	4O5L	4YAQ	5I18
1GAF	2ZKH	3QCT	4HII	4OB5	4YHI	5I1D
1HKL	3AAZ	3QCU	4HIJ	4OCR	4YHK	5I8K
1HZH	3EO9	3QCV	4HK3	4OCS	4YHL	5I8O
1JPT	3EYO	3QHF	4HQQ	4OCW	4YHM	5IFA
1JV5	3EYQ	3QHZ	4HWE	4OD1	4YHO	
1OHQ	3F12	3QO1	4JAM	4OD3	4YHY	
1OPG	3FCT	3QYC	4JDV	4ODH	4ZD3	
1R70	3GIZ	3TCL	4JFY	4OSU	4ZYK	
1RZ7	3GJE	3U36	4JHA	4P9M	5ALB	
1RZF	3GKW	3U46	4JY4	4PTT	5ALC	
1RZG	3HC0	3U4B	4JY5	4PTU	5AWN	
1RZI	3HC3	3U6R	4K3G	4PUB	5AZE	
1U6A	3HC4	3W9D	4K3H	4QHM	5BMF	
1WT5	3HI5	3X3G	4KMT	4QHN	5BQ7	
1ZVO	3HMW	3ZL4	4KQ3	4QXG	5CCK	
2AGJ	3INU	4DN3	4KY1	4R26	5CD3	
2D7T	3KDM	4F57	4LKC	4R7D	5CEY	

Supplementary Table 3. Binding Region and Mutation Patterns of aAb

**PDBID**	**Binding region***	**epi_bg_mutation[a]**	**epi_bg_insertion[b]**	**epi_bg_same[c]**
**1AD9**	Fab 2	Y		
**1ADQ**	Fc			
**1RHH**	Fab 2			Y
**1RZ8**	V	Y	Y	
**1ZA6**	V	Y	Y	
**2A9M**	V	Y		
**2A9N**	V	Y		
**2AJ3**	Fab 2	Y		
**2AJ3**	Fab 2	Y	Y	
**2FJF**	V	Y	Y	
**2FJF**	V	Y		
**2FL5**	Fab 2	Y		
**2J6E**	Fc			
**2J6E**	Fc			
**3D69**	V			
**3DGG**	V	Y	Y	
**3DIF**	V	Y	Y	
**3G6A**	Fab 2	Y		
**3KYM**	V	Y	Y	
**3L7F**	V	Y	Y	
**3L7F**	V	Y		

**3MUG**	Fab 2	Y		
**3MUG**	Fab 2	Y		
**4EVN**	V	Y	Y	
**4FQH**	V	Y		
**4FQQ**	V	Y	Y	
**4HH9**	V	Y		
**4HK0**	V	Y		
**4HKB**	V	Y		
**4JPI**	V	Y		
**4JY6**	Fab 2			Y
**4LLV**	V	Y	Y	
**4LLV**	V	Y	Y	
**4LLV**	V	Y	Y	
**4M5Y**	V	Y	Y	
**4NM4**	V	Y		
**4ORG**	V	Y	Y	
**4PY7**	V	Y		
**4QHK**	V	Y	Y	
**4QHL**	V	Y		
**4R4B**	V	Y		
**4X0K**	V	Y	Y	
**4XBG**	V	Y		
**4XBG**	V	Y		
**4XBP**	V	Y	Y	
**4XCE**	V		Y	
**4XCE**	V	Y	Y	
**4XCN**	V	Y	Y	
**4XCY**	V	Y	Y	
**4XCY**	V	Y		
**4YGV**	V	Y	Y	
**5ANM**	C			
**5BVJ**	V	Y		
**5CEX**	V	Y	Y	

*C means Fc, V means Fv and Fab 2 means non-CDR Fab.

[a]Y means this aAb contains residue mutations compared with background antibodies in CDR

[b]Y means this aAb contains residue insertion compared with background antibodies in CDR

[c]Y means this aAb contains same CDR residue composition compared with background antibodies.
